# IMPROVING GLUTATHIONE, MITOCHONDRIA, INFLAMMATION, AND COGNITIVE DECLINE: A PILOT CLINICAL TRIAL OF GLYNAC IN AGING

**DOI:** 10.1093/geroni/igae098.0809

**Published:** 2024-12-31

**Authors:** Rajagopal Sekhar, Premranjan Kumar, Chun Liu, Jean Hsu, Chacko Shaji, Jahoor Farook, Minard Charles

**Affiliations:** Baylor College of Medicine, Houston, Texas, United States; Baylor College of Medicine, Houston, Texas, United States; Baylor College of Medicine, Houston, Texas, United States; Baylor College of Medicine, Houston, Texas, United States; Baylor College of Medicine, Houston, Texas, United States; Baylor College of Medicine, Houston, Texas, United States; Baylor College of Medicine, Houston, Texas, United States

## Abstract

Although aging is associated with cognitive decline, mechanisms contributing to ‘age-related cognitive decline’ (ACD) are not well understood. Aging is associated with glutathione deficiency, oxidative stress, mitochondrial dysfunction, inflammation, insulin resistance and endothelial function (many of these identified as aging hallmarks and are key defects in Geroscience), and these defects could compromise lead to cognitive impairment. Based on prior translational studies in aging where we found evidence that supplementing GlyNAC (combination of glutathione precursors glycine and N-acetylcysteine) could improve glutathione deficiency, oxidative stress, mitochondrial dysfunction and glucose tolerance, we conducted a 36-week pilot human clinical trial comparing young-adults (YA) and older-adults (OA). We found that compared to YA, OA had evidence of cognitive decline together with glutathione deficiency, oxidative stress, mitochondrial dysfunction, inflammation, insulin resistance and endothelial dysfunction and deficiency of BDNF (brain-derived neurotropic factor). Supplementing GlyNAC for 24-weeks reversed these defects and improved cognition, but stopping GlyNAC for 12-weeks led to redevelopment of these defects. Using tracer methodologies we also identified a novel ‘brain glucose steal’ phenomenon wherein, due to mitochondrial impairment, non-brain organs were found to be utilizing glucose intended for the brain, and GlyNAC supplementation led to reversal of the ‘brain glucose steal’ phenomenon. Collectively, the findings from this trial provide proof-of-concept supporting GlyNAC supplementation in OA to improve multiple mechanistic defects related to cognitive impairment, and thereby also cognitive function and brain health. As many of these defects also occur in human Alzheimer’s disease (AD), these findings also have implications for improving brain health in AD.

